# Plasma antioxidant capacity in critical traumatized patients: severity and anatomical location

**DOI:** 10.1186/cc14386

**Published:** 2015-03-16

**Authors:** G Papakitsos, A Kapsali, T Papakitsou, A Roimba

**Affiliations:** 1 GHA, Arta, Greece; 2 GHM, Messologi, Greece; 3 General Practitioner, Thessaloniki, Greece

## Introduction

Oxidative stress (OS) has been invoked as a relevant factor in the evolution and outcome of critical care patients. Indeed, antioxidant therapies have been used in critical care patients, but with controversial results. This may be explained by assuming OS as a homeostatically regulated parameter and both its excess and its deficit influencing severity progression. Nonetheless, antioxidant agents could mask an OS signaling role, blocking otherwise physiological responses aimed at recovery of homeostasis. We have evaluated plasma total antioxidant capacity (TAC) in traumatized patients in the emergency department (ED) and we determined its potential relationship with severity and trauma location.

## Methods

In a prospective observational study of ED polytraumatized patients (*n = *23, mean Acute Physiology and Chronic Health Evaluation II (APACHE II) score of 11 ± 6) we measured (in the first 24 hours) plasma TAC by the ferric reducing activity/antioxidant power (FRAP). For control subjects, we used age-matched and gender-matched volunteers (*n *= 32). We also evaluated the contribution of antioxidant molecules (uric acid, bilirubin, and albumin) to these values.

## Results

Polytraumatized patients show differences in TAC with reference to control subjects. ED polytraumatized patients show high FRAP values. We found that FRAP values were inversely correlated with APACHE II score (*r *= -0.266, *P *< 0.01) suggesting that, in trauma patients, increased antioxidant response, as measured by FRAP assay, could be a pathophysiological response to stress. Albumin and uric acid concentrations reproduced the FRAP trend with severity.

## Conclusion

FRAP values in trauma ED patients are independently influenced by age (β = 0.271, *P *< 0.021), APACHE II score (β = -0.356, *P *< 0.002) and head trauma (β = -0.219, *P *< 0.045). These results accentuate the influence of trauma location and severity in TAC changes. TAC response in ED patients reinforces the need for an adequate tailoring of treatments aimed at their recovery, such as antioxidant therapies.

